# Musical Expertise Modulates Early Processing of Syntactic Violations in Language

**DOI:** 10.3389/fpsyg.2012.00603

**Published:** 2013-01-11

**Authors:** Ahren B. Fitzroy, Lisa D. Sanders

**Affiliations:** ^1^Neuroscience and Behavior Program, University of MassachusettsAmherst, MA, USA; ^2^Department of Psychology, University of MassachusettsAmherst, MA, USA

**Keywords:** expertise, syntax, music, language, sentence processing, ERAN, LAN, P600

## Abstract

Syntactic violations in speech and music have been shown to elicit an anterior negativity (AN) as early as 100 ms after violation onset and a posterior positivity that peaks at roughly 600 ms (P600/LPC). The language AN is typically reported as left-lateralized (LAN), whereas the music AN is typically reported as right-lateralized (RAN). However, several lines of evidence suggest syntactic processing of language and music rely on overlapping neural systems. The current study tested the hypothesis that syntactic processing of speech and music share neural resources by examining whether musical proficiency modulates ERP indices of linguistic syntactic processing. ERPs were measured in response to syntactic violations in sentences and chord progressions in musicians and non-musicians. Violations in speech were insertion errors in normal and semantically impoverished English sentences. Violations in music were out-of-key chord substitutions from distantly and closely related keys. Phrase-structure violations elicited an AN and P600 in both groups. Harmonic violations elicited an LPC in both groups, blatant harmonic violations also elicited a RAN in musicians only. Cross-domain effects of musical proficiency were similar to previously reported within-domain effects of linguistic proficiency on the distribution of the language AN; syntactic violations in normal English sentences elicited a LAN in musicians and a bilateral AN in non-musicians. The late positivities elicited by violations differed in latency and distribution between domains. These results suggest that initial processing of syntactic violations in language and music relies on shared neural resources in the general population, and that musical expertise results in more specialized cortical organization of syntactic processing in both domains.

## Introduction

Measuring the effects of expertise in one domain on processing in another domain provides a way to define shared neural resources. To the extent that processing in two domains relies on the same neural tissue, cortical reorganization in the domain of expertise should be accompanied by reorganization of processing in the other domain. When this is true, the neural resource can be considered domain-general in that it is capable of contributing to processing in multiple domains. Language and music are ideal for testing the effects of expertise on cortical organization. All typically functioning adults have extensive experience with at least one language. However, they are expected to have more variable levels of experience with music. Further, language and music are both systems of communication in which discrete units are organized according to rules. During speech processing, speakers of a language apply the structural rules they are familiar with to extract information. Like languages, musical systems contain rules of structural arrangement such that within a prevailing context certain events can be viewed as ungrammatical. The Western tonal musical system contains rules regarding the temporal arrangement and stress patterning of musical events (Lerdahl and Jackendoff, 1983), as well as the arrangement of pitches in monophonic (melody) and polyphonic (harmony) phrases (Krumhansl and Shepard, 1979; Krumhansl and Kessler, 1982; Bharucha and Krumhansl, 1983). Together these rules can be considered the grammar of Western music, and they are applied in a hierarchical and interactive fashion to a limited number of base musical units to form an effectively unlimited set of expressions (Lerdahl and Jackendoff, 1983).

Although there are clearly differences in the structures of linguistic and musical grammars, recent evidence suggests similarities in syntactic processing of language and music (for review, see Patel, 2008). Similarities in event-related potential (ERP) indices of linguistic and harmonic syntactic processing raise the possibility that syntactic processing in the two domains is subserved by shared neural resources (Patel et al., 1998; Koelsch et al., 2000; Patel, 2003). Further, simultaneous presentation of syntactic violations in language and music affects ERP indices of linguistic syntactic processing (Koelsch et al., 2005). Musical expertise has been shown to modulate the processing of non-syntactic aspects of speech, including metric structure and pitch (Schön et al., 2004; Marques et al., 2007; Kraus and Chandrasekaran, 2010; Besson et al., 2011; Marie et al., 2011). Recent ERP evidence suggests that musical expertise also affects the developmental time-course of linguistic syntactic processing (Jentschke and Koelsch, 2009). Specifically, musically trained compared to untrained 10- and 11-year-old children showed a more adult-like ERP response to linguistic syntactic violations. These results could represent a qualitative difference in linguistic syntactic processing as a function of musical expertise, but alternatively may reflect speeded development of linguistic syntactic processing in musically trained children. To differentiate between these two interpretations, it is necessary to examine the effects of musical expertise on linguistic syntactic processing in adults.

There are well-established ERP indices of syntactic processing in both language and music. The first part of the response to syntactic violations in language is an anterior negativity (AN) 100–500 ms post violation onset, typically reported as left-lateralized (LAN). The LAN has been observed in response to a range of syntactic violations, including word category rule violations (Neville et al., 1991; Friederici et al., 1993). The LAN is thought to index an automatic parsing mechanism since it is unaffected by the proportion of violations (Hahne and Friederici, 1999). The extent to which semantic content affects syntactic processing has been explored to distinguish between syntax-first and interactive models of speech processing. One approach has been to compare ERP responses to syntactic violations in normal sentences and in Jabberwocky sentences in which all open-class words are replaced by pseudowords (Münte et al., 1997; Canseco-Gonzalez, 2000; Hahne and Jescheniak, 2001; Yamada and Neville, 2007). The results of these experiments are mixed; syntactic violations in semantically impoverished sentences elicit a LAN. However, two of the three studies that observed a LAN with both semantically intact and impoverished sentences found differences in distribution between conditions (Canseco-Gonzalez, 2000; Yamada and Neville, 2007). Even with normal sentences, there are differences in the distribution of the LAN across studies. Linguistic syntax violations elicited LANs in a number of studies (e.g., Neville et al., 1991; Münte et al., 1993; Hahne and Friederici, 1999), but a broadly distributed or bilateral AN in many studies as well (e.g., Hahne, 2001; Hahne and Friederici, 2002; Hagoort et al., 2003; for full list, see Pakulak and Neville, 2010). A key recent finding suggests that this inconsistency is in part due to differences in linguistic proficiency among participants (Pakulak and Neville, 2010). Higher proficiency monolingual speakers show a more focal, left-lateralized AN than lower proficiency individuals.

The second part of the response to syntactic violations in language is a posterior positivity that peaks roughly 600 ms post violation onset (P600 or syntactic positive shift, SPS). The P600 is elicited by ungrammatical events including phrase-structure violations (Neville et al., 1991; Friederici et al., 1993; Hagoort et al., 1993; Münte et al., 1997), but is also observed in response to grammatical but less preferred constructions (Osterhout and Holcomb, 1992; Osterhout et al., 1994; Hagoort et al., 1999; Hagoort and Brown, 2000; Kaan et al., 2000; Friederici et al., 2002; Kaan and Swaab, 2003; for review, see Gouvea et al., 2010). The amplitude of the P600 has been suggested to index the amount of resources used to integrate the critical word with the current model of sentence structure (Kaan et al., 2000; Kaan and Swaab, 2003). Supporting this argument, a larger P600 was observed for disambiguating words in sentences of greater complexity (Kaan et al., 2000; Friederici et al., 2002; Kaan and Swaab, 2003) and for ungrammatical compared to less preferred words (Kaan and Swaab, 2003). Syntactic violations in Jabberwocky sentences result in a smaller or even absent P600 (Münte et al., 1997; Canseco-Gonzalez, 2000; Yamada and Neville, 2007; but see also Hahne and Jescheniak, 2001). Kaan et al. (2000) argue that the reduction in P600 amplitude reflects fewer resources used when fewer features can be integrated. Further, proficiency is positively correlated with P600 amplitude in monolinguals (Pakulak and Neville, 2010). This effect could represent either greater recruitment of the resources all individuals have for syntactic integration or additional integration resources that are only available in experts.

Similar to findings in the language domain, violations of musical syntax have been shown to elicit an AN. Violations of Western harmonic norms elicit an AN 150–400 ms post onset that is typically described as right-lateralized (RAN; Patel et al., 1998; Koelsch et al., 2000; for review, see Koelsch, 2009). The RAN was first reported in a later time window (300–400 ms) with an anterior-temporal distribution (Patel et al., 1998), but most subsequent studies show the RAN in an earlier time window (150–300 ms, ERAN) with a more focally anterior distribution (Koelsch et al., 2000, 2002a,b, 2003, 2005; Loui et al., 2005; Leino et al., 2007; Koelsch and Jentschke, 2008; Koelsch and Sammler, 2008; Steinbeis and Koelsch, 2008). The latency and distribution differences may be driven by subtle differences in the stimuli including the presence of rhythmic patterning, phrase length, and the type of harmonic incongruity (Koelsch, 2009). As is true for language, the lateralization of the AN elicited by violations in music is not entirely consistent. Many studies show an AN that is clearly right-lateralized (Patel et al., 1998; Koelsch et al., 2000, 2002a, 2007; Koelsch and Jentschke, 2008; Koelsch and Sammler, 2008; Steinbeis and Koelsch, 2008), but there are multiple reports of broadly distributed or bilateral ANs (Loui et al., 2005; Steinbeis et al., 2006; Leino et al., 2007; Miranda and Ullman, 2007; Koelsch and Jentschke, 2010; Villarreal et al., 2011). Proficiency does not appear to explain the differences in lateralization for music. Right-lateralized and bilateral ANs have been observed in non-musicians, amateur musicians, and expert musicians (e.g., Koelsch et al., 2002a; Steinbeis et al., 2006; Miranda and Ullman, 2007; Koelsch and Jentschke, 2008). However, musicians show larger amplitude ANs than non-musicians in response to music syntactic violations (Koelsch et al., 2002a, 2007).

Harmonic incongruities in unfamiliar melodies and chord progressions also elicit a late positive component (LPC) maximal 300–800 ms post onset that is broadly distributed across posterior regions (Besson and Faïta, 1995; Janata, 1995; Patel et al., 1998; Koelsch et al., 2000; Regnault et al., 2001; Koelsch and Mulder, 2002; Brattico et al., 2006; Miranda and Ullman, 2007; Carrión and Bly, 2008; Peretz et al., 2009). The LPC is larger in amplitude when elicited by mistuned compared to non-diatonic notes (Peretz et al., 2009), by non-diatonic compared to diatonic incongruities (Besson and Faïta, 1995; Brattico et al., 2006), by less harmonically expected phrase-final chords (Janata, 1995; Carrión and Bly, 2008), and by more distantly related phrase-internal out-of-key chords (Patel et al., 1998). These findings have been taken to suggest that the LPC indexes the integration of an unexpected note or chord into the current harmonic context, a function similar to that indexed by the P600 elicited by linguistic syntactic violations (Patel et al., 1998; Koelsch, 2011; but see also Regnault et al., 2001). The effects of musical expertise on the LPC are unclear; proficiency has been reported to modulate LPC amplitude under some conditions (Besson and Faïta, 1995), but not others (Regnault et al., 2001; Miranda and Ullman, 2007). Using a within-subjects design, Patel et al. (1998) found no significant amplitude or latency differences between the late positivities elicited by out-of-key chord substitutions and linguistic phrase-structure violations, suggesting that these ERP effects are driven by the same domain-general process.

Similarities between ERP indices of syntactic processing in language and music have contributed to the *shared syntactic integration resource hypothesis* (SSIRH), which posits that the integration of syntactic structure relies on domain-general neural resources (Patel, 2003). SSIRH assumes a limited capacity syntactic processing mechanism and predicts that simultaneous processing of linguistic and musical syntax will cause interference (Patel, 2003). This prediction is tentatively supported by evidence that simultaneous presentation of linguistic and harmonic violations results in a reduced LAN (Koelsch et al., 2005; Steinbeis and Koelsch, 2008) and RAN under some conditions (Steinbeis and Koelsch, 2008; Maidhof and Koelsch, 2011). The prediction is further supported by behavioral evidence; harmonic violations increase reading times for difficult to integrate words in garden-path sentences, decrease comprehension of garden-path sentences and complex object-first relative clauses, and reduce the processing facilitation observed for expected words (Fedorenko et al., 2009; Slevc et al., 2009; Hoch et al., 2011). Further, the cross-domain interference can be shown to be selectively syntactic. No interference was found between harmonic violations and semantic processing (Slevc et al., 2009), or between auditory deviance and syntactic complexity (Fedorenko et al., 2009).

The current study employed a within-subjects design and ERP measures to test whether musical expertise modulates cortical organization of linguistic syntactic processing in adults. Musically trained and untrained participants listened to spoken sentences and chord progressions with and without syntactic violations and judged the correctness of each phrase. Phrase-structure violations were presented in English and Jabberwocky. Harmonic violations were out-of-key chord substitutions from distant and nearby keys. Syntactic violations were expected to elicit an AN and a late positivity in both groups, replicating previous ERP studies of either language or music processing. The syntactic violations in Jabberwocky sentences and chord substitutions from nearby keys were expected to elicit a reduced late positivity (P600/LPC). The within-subjects design was expected to reveal an AN that differed in distribution between domains, but a late positivity that did not. The effects of musical expertise on within-domain syntactic processing were predicted to result in a larger AN and LPC to harmonic violations in musicians. Critically, musical expertise was also predicted to affect ERP indices of linguistic syntactic processing in a manner similar to linguistic proficiency such that phrase-structure violations would elicit a more focal and left-lateralized AN and a larger P600 in musicians.

## Materials and Methods

### Participants

Forty adults provided the data included in analysis. All participants were right-handed, native English speakers with normal hearing, normal or corrected to normal vision, and no known neurological conditions. Participants were divided into two groups on the basis of their self-reported musical training and performance backgrounds; 20 (nine female) were classified as musicians and 20 (seven female) as musically naïve members of the general population (subsequently referred to as “non-musicians”). Data from an additional seven participants were excluded from analyses due to artifacts in electroencephalogram (EEG), insufficiently high or low levels of musical expertise, or poor performance on the behavioral task.

The musicians were ages 19–28 years (*M* = 21.5, SD = 2.4). Four musicians reported having 3–5 years of experience on their primary instrument, seven reported 6–9 years, and eight reported 10 or more years. All musicians had prior music theory training, with 13 having completed Music Theory II or above at the college level. The non-musicians were ages 18–49 years (*M* = 25.0, SD = 10.1). Of the non-musicians who had any experience with an instrument, three reported having less than 1 year on their primary instrument, seven reported having 1–2 years, five reported having 3–5 years, and one reported having 6–9 years. Only two non-musicians had any prior music theory training, which was at or below the high school level.

### Materials

Prior to data collection, participants completed a questionnaire designed to classify level of musical expertise based on years of instrument performance, formal musical training, and music listening habits as children and adults.

The 240 sentences consisted of 60 sets of the same sentence recorded in the four forms shown in Table 1. The sentences in the current experiment were a subset of those employed by Yamada and Neville (2007) and Pakulak and Neville (2010). All sentences were recorded by the same female speaker and were less than 3 s in length. The syntactic violations were insertion errors in which a closed-class word was inserted prior to one of the grammatically correct closed-class words in the sentence. This type of violation allows for comparing ERP responses to the same physical stimulus (e.g., “her”) in canonical and violation contexts.

**Table 1:** **Examples of speech stimuli**.

*English canonical:* Kara can write the letters to her friends.
*English violation:* Kara can write the letters to those her friends.
*Jabberwocky canonical:* Cooly can wrog the lapples to her floams.
*Jabberwocky violation:* Cooly can wrog the lapples to those her floams.

**Figure 1:**
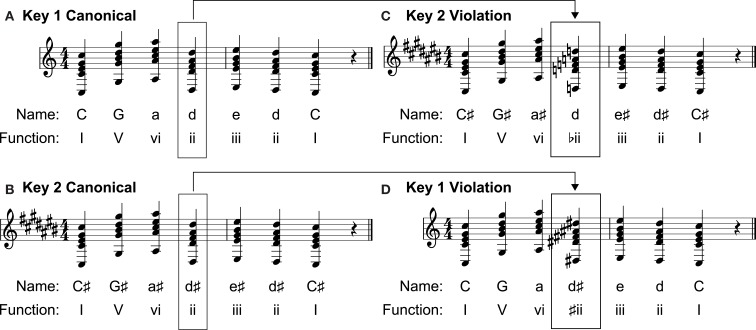
**Music stimuli design**. Canonical musical stimuli for the blatant condition were created by spelling out a relative chord progression in two keys separated by five degrees on the circle of fifths, such as C **(A)** and C♯ **(B)**. The fourth, fifth, or sixth chords in the progressions were then switched to create blatant harmonic violations such as those shown in **(C)** and **(D)**.

The 240 musical sequences consisted of short, isochronous (142 bpm) phrases composed in major keys according to typical rules of Western tonal harmony. As shown in Figure 1, each phrase was a seven-chord progression. All chords were triads with the bass note repeated as high and low voices. All critical comparison chords were in root position; the majority of the non-critical chords were also in root position, but some were presented in first or second inversion. Each progression started and ended with the tonic chord of the intended major key. Half of the phrases included one out-of-key chord substitution similar to those employed by Patel et al. (1998), equally likely in the fourth, fifth, or sixth position. Of those chord substitutions, half were from distantly related keys creating blatant harmonic violations and half were from closely related keys creating more subtle harmonic violations. In Western tonal music, keys are perceived as more closely or distantly related to one another as a function of their distance on the circle of fifths (Krumhansl and Kessler, 1982; Patel et al., 1998). Out-of-key chord substitutions from distantly related keys are more often identified as unacceptable than substitutions from closely related keys (Patel et al., 1998). Music stimuli were composed using MIDI authoring software (Cakewalk Home Studio 2004) and generated by an external MIDI synthesizer (Yamaha DGX-202).

In the blatant conditions, 60 phrases were created by spelling out 10 chord progressions in three pairs of keys separated by five degrees on the circle of fifths (Appendix – Musical items). One chord was then substituted into the same position in the other progression in a pair to create the blatant violations (Figure 1). This substitution makes it possible to compare ERPs for the same stimulus in canonical and violation contexts. The quality and function of the chords at the critical position varied: I, ii, iii, IV, V, vi, and vii° all occurred as critical chords. In each pair of phrases, the chords used to create the violations served the same relative function in the canonical conditions and served as altered versions with all notes raised or lowered by one half-step in the violation conditions. The ungrammatical chords in the blatant violation condition contained one to three out-of-key notes. In the subtle conditions, 60 canonical phrases were created as 30 pairs of progressions in keys separated by three degrees on the circle of fifths (Appendix – Musical items). Fifteen of the pairs were composed such that if a subdominant (IV) chord occurred at the critical position in one progression, a supertonic (ii) chord occurred at the same position in the other. The other 15 pairs were composed such that if a dominant (V) chord occurred at the critical position in one progression, a mediant (iii) chord occurred at the same position in the other. The chords at the critical position of each progression were then switched to create the 60 violation phrases. The result of this manipulation is that the substituted chords retained the root and function of the chord being replaced, but the quality of the critical chord changed from major to minor or from minor to major. The ungrammatical chords in this condition contained one out-of-key note.

### Procedure

All procedures were approved by the University of Massachusetts, Amherst Institutional Review Board. Participants provided informed consent, completed the music-experience questionnaire, and answered demographic questions before being fitted with an electrode net. EEG was collected using a 128-channel net (EGI, Eugene, OR, USA) at a sampling rate of 250 Hz with a bandpass of 0.1–100 Hz. Participants were instructed to listen to the sentences and chord progressions and to press a button after each to indicate if the phrase sounded correct or if something was strange or out of place. Participants completed practice trials and were encouraged to ask questions. Participants were asked to maintain fixation during sound presentation. When the fixation point disappeared, participants provided their judgment of correctness then pressed any button to begin the next trial. All of the music and speech stimuli were presented in the same pseudo-randomized order for every participant. The experiment lasted 2 h, and participants were given opportunities for breaks every 15 min.

Continuous EEG was segmented into 1200 ms epochs beginning 200 ms before the target word or chord. Epochs containing artifacts defined by maximum amplitude criteria established for each participant by observing the effects of eye-movements, blinks, and other motions on EEG during instructions were excluded from analyses. Artifact-free epochs from trials on which participants gave correct behavioral responses were averaged separately for each participant and condition. One musician did not provide a sufficient number of correct trials in the Jabberwocky condition, so data from this participant were excluded from all ERP comparisons involving Jabberwocky stimuli. Averaged waveforms were re-referenced to the average of the two mastoid recordings and baseline corrected to the 100 ms prior to target onset.

### Analyses

Performance was assessed by calculating *d*′ separately for English sentences, Jabberwocky sentences, canonical chord progressions and those with the matched blatant violations, and canonical chord progressions and those with the matched subtle violations.

**Figure 2:**
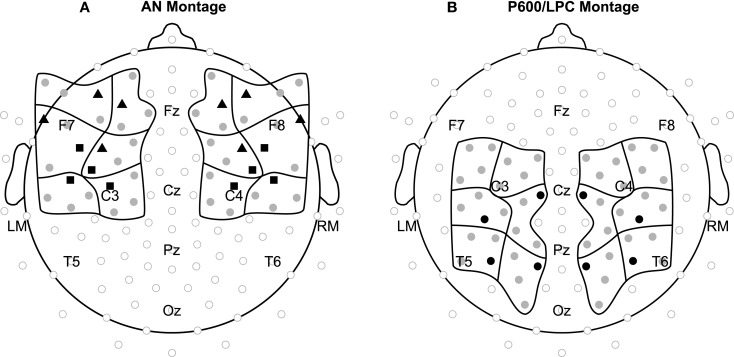
**Electrode montages**. Electrode positions included in statistical analyses for the *(A)* AN and *(B)* P600/LPC are shown in black and gray grouped into 12 regions of interest. Waveforms shown in the Language results (Figure 4) and Music results (Figure 5) sections are from electrodes shown in black (triangles = language AN, squares = music AN, circles = language P600 and music LPC).

To best capture the anterior ERP effects, 12 regions of interest were established by averaging data across four electrodes. The 12 regions varied in anterior/posterior position (AP: three levels), lateral/medial position (LAT: two levels), and hemisphere (HEM: two levels) as shown in Figure 2. For language stimuli, mean amplitude at these electrodes was measured 100–250 ms (early AN) and 300–500 ms (late AN). For music stimuli, mean amplitude at these electrodes was measured 150–300 ms. To determine if the onset latency of effects observed for language and music differed, additional mean amplitude measures were taken 100–150 ms. To best capture the posterior effects, 12 new regions of interest were established by averaging data across four electrodes. These regions also varied in anterior/posterior (three levels), lateral/medial (two levels), and hemisphere (two levels) position (see Figure 2). Mean amplitude at these electrodes was measured 200–750 ms for language stimuli and 400–950 ms for music stimuli. To compare onset latencies, mean amplitude was also measured 200–400 ms for both domains.

To fully define the effects of grammaticality on ERPs, each mean amplitude measure was initially subjected to a Greenhouse–Geisser corrected, repeated-measures ANOVA: CV (canonical, violation) × AP × LAT × HEM separately for each group and stimulus set. Initial CV × AP interactions motivated limiting the analysis of the early language AN to the two anterior-most rows of electrodes, the late language AN to the single most anterior row, and the music AN to the two central-most rows. Additional analyses were conducted to determine if amount of semantic content in sentences (SEM: English, Jabberwocky), distance between the keys of the context and substituted chords (DIS: blatant, subtle), group (GP: non-musician, musician), or domain (DOM: language, music) modulated the grammaticality effects.

## Results and Discussion

### Language results

#### Language behavioral results

**Figure 3:**
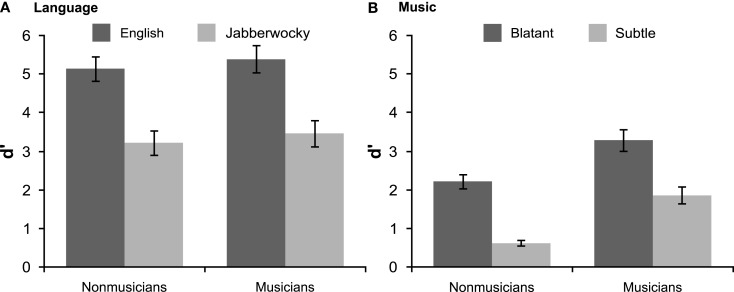
**Behavioral results**. Performance on the grammaticality judgment task is shown for **(A)** language and **(B)** music with standard error bars.

Both groups of participants detected phrase-structure violations and showed better performance with English than Jabberwocky (Figure 3). Non-musicians’ performance was above chance for both types of sentences [English: *d*′ = 5.14, *SD* = 1.44, *t*(19) = 15.94, *p* < 0.001; Jabberwocky: *d*′ = 3.22, *SD* = 1.43, *t*(19) = 10.09, *p* < 0.001] and better for English, *t*(19) = 5.54, *p* < 0.001. Similarly, musicians’ performance was above chance for both types of sentences [English: *d*′ = 5.39, *SD* = 1.58, *t*(19) = 15.23, *p* < 0.001; Jabberwocky: *d*′ = 3.46, *SD* = 1.54, *t*(19) = 10.03, *p* < 0.001] and better for English, *t*(19) = 6.79, *p* < 0.001. There was no evidence that musical expertise modulated ability to detect phrase-structure violations (*p*s > 0.5).

#### English ERP results

**Figure 4:**
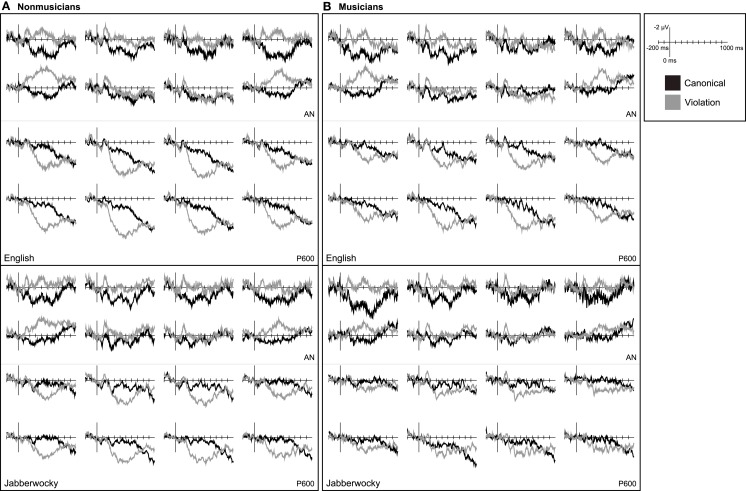
**Language ERPs**. Grand average ERP waveforms elicited by critical words in canonical and violation contexts measured in **(A)** non-musicians and **(B)** musicians. Data for English sentences are shown on the top and for Jabberwocky sentences on the bottom.

In non-musicians correctly-detected violations in English sentences elicited a bilateral AN and a later posterior positivity (Figure 4). The early AN 100–250 ms after onset was observed across all regions of interest, *F*(1, 19) = 7.66, *p* = 0.012. As was true for all subsequent comparisons of mean amplitude in this time window, a grammaticality by anterior/posterior interaction, *F*(2, 38) = 34.49, *p* < 0.001, indicated that the AN was larger over more anterior regions [most anterior row CV: *F*(1, 19) = 17.84, *p* < 0.001; second-most anterior row CV: *F*(1, 19) = 11.70, *p* = 0.003; two anterior-most rows CV: *F*(1, 19) = 15.54, *p* = 0.001]. The late AN 300–500 ms after onset was only observed over the most anterior regions [all regions CV × AP: *F*(2, 38) = 64.23, *p* < 0.001; most anterior row CV: *F*(1, 19) = 13.91, *p* = 0.001; second-most anterior row CV: *F*(1, 19) = 1.27, *p* = 0.273]. The late AN was larger at lateral sites [most anterior row CV × LAT: *F*(1, 19) = 34.50, *p* < 0.001]. There was no evidence that the negativity in either time window was lateralized to the left or right-hemisphere (*p*s > 0.1). These violations also elicited a posterior positivity 200–750 ms, *F*(1, 19) = 17.11, *p* = 0.001, that was largest over more posterior and medial regions [CV × AP: *F*(2, 38) = 27.53, *p* < 0.001; CV × LAT: *F*(1, 19) = 45.94, *p* < 0.001; CV × AP × LAT: *F*(2, 38) = 5.52, *p* = 0.010; CV × AP × HEM: *F*(2, 38) = 7.92, *p* = 0.006].

In musicians correctly-detected violations in English sentences elicited a LAN and a later posterior positivity (Figure 4). The early AN 100–250 ms after onset was larger over more anterior regions [all regions CV: *F*(1, 19) = 8.96, *p* = 0.007 and CV × AP: *F*(2, 38) = 37.52, *p* < 0.001; two anterior-most rows CV: *F*(1, 19) = 13.73, *p* = 0.002]. The early AN was left-lateralized [two anterior-most rows CV × HEM: *F*(1, 19) = 5.96, *p* = 0.025] and larger at lateral sites [two anterior-most rows CV × LAT: *F*(1, 19) = 6.14, *p* = 0.023]. Although the early AN was larger over the left-hemisphere, it was evident at left electrodes alone [two anterior-most rows left CV: *F*(1, 19) = 23.99, *p* < 0.001] and at right electrodes alone [two anterior-most rows right CV: *F*(1, 19) = 5.68, *p* = 0.028]. The late AN 300–500 ms after onset was observed over the most anterior regions [all electrodes CV × AP: *F*(2, 38) = 98.54, *p* < 0.001; most anterior row CV: *F*(1, 19) = 16.16, *p* = 0.001]. In musicians, the late AN was also left-lateralized [most anterior row CV × HEM: *F*(1, 19) = 4.53, *p* = 0.047] and larger at lateral sites [most anterior row CV × LAT: *F*(1, 19) = 23.12, *p* < 0.001]. The late AN was evident at left electrodes alone [most anterior row left CV: *F*(1, 19) = 21.71, *p* < 0.001] and at right electrodes alone [most anterior row right CV: *F*(1, 19) = 8.12, *p* = 0.010]. These violations also elicited a posterior positivity 200–750 ms, *F*(1, 19) = 12.96, *p* = 0.002, that was largest over more posterior and medial regions [CV × AP: *F*(2, 38) = 18.54, *p* < 0.001; CV × LAT: *F*(1, 19) = 43.28, *p* < 0.001]. The posterior positivity in response to violations in English sentences was right-lateralized in musicians [CV × HEM: *F*(1, 19) = 9.04, *p* = 0.007; CV × AP × HEM: *F*(2, 38) = 13.36, *p* < 0.001].

#### Jabberwocky ERP results

In non-musicians correctly-detected violations in Jabberwocky sentences elicited a bilateral AN and a later posterior positivity (Figure 4). The early AN 100–250 ms after onset was larger at more anterior sites [all regions CV: *F*(1, 19) = 14.46, *p* = 0.001 and CV × AP: *F*(2, 38) = 15.70, *p* < 0.001; two anterior-most rows CV: *F*(1, 19) = 19.32, *p* < 0.001]. There was no evidence that the early AN was left- or right-lateralized (*p*s > 0.1) or differed from what was observed for English (*p*s > 0.2). The late AN 300–500 ms after onset was observed over the most anterior regions [all regions CV × AP: *F*(2, 38) = 64.82, *p* < 0.001; most anterior row CV: *F*(1, 19) = 15.72, *p* = 0.001]. As was true for English sentences, the later AN was larger at lateral sites [most anterior row CV × LAT: *F*(1, 19) = 11.83, *p* = 0.003]. However, there was some suggestion that the difference in the later AN measured over lateral and medial regions was larger for English sentences [SEM × CV × LAT: *F*(1, 19) = 4.27, *p* = 0.053]. Syntactic violations in Jabberwocky sentences also elicited a posterior positivity 200–750 ms after onset, *F*(1, 19) = 9.39, *p* = 0.006, that was largest over posterior and medial regions [CV × AP: *F*(2, 38) = 22.12, *p* < 0.001; CV × LAT: *F*(1, 19) = 31.45, *p* < 0.001; CV × AP × LAT: *F*(2, 38) = 8.85, *p* = 0.001; CV × AP × HEM: *F*(2, 38) = 3.97, *p* = 0.037]. In non-musicians there was no evidence that the posterior positivity elicited by syntactic violations differed between English and Jabberwocky (*p*s > 0.2).

In musicians correctly-detected violations in Jabberwocky sentences elicited an AN and a later posterior positivity (Figure 4). The early AN 100–250 ms after onset was larger over more anterior regions [all regions CV: *F*(1, 18) = 13.13, *p* = 0.002 and CV × AP: *F*(2, 36) = 19.02, *p* < 0.001; two anterior-most rows CV: *F*(1, 18) = 17.33, *p* = 0.001]. Although the early AN in musicians listening to Jabberwocky was not left-lateralized, there was also no evidence that it differed from the left-lateralized effect observed with English (*p*s > 0.3) except that the difference at lateral and medial sites was larger with English [SEM × CV × LAT: *F*(1, 18) = 7.26, *p* = 0.015]. The later AN 300–500 ms after onset was observed at more anterior sites [all regions CV × AP: *F*(2, 36) = 73.60, *p* < 0.001; most anterior row CV: *F*(1, 18) = 12.85, *p* = 0.002]. The later AN was larger at lateral sites [most anterior row CV × LAT: *F*(1, 18) = 10.17, *p* = 0.005] and did not differ in amplitude or distribution from that observed with English (*p*s > 0.2). Violations in Jabberwocky sentences also elicited a posterior positivity 200–750 ms after onset, *F*(1, 18) = 4.83, *p* = 0.041. This effect was larger over more posterior and medial sites [CV × AP: *F*(2, 36) = 10.07, *p* = 0.003; CV × AP × LAT: *F*(2, 36) = 5.95, *p* = 0.006]. The posterior positivity had a more medial distribution for English compared to Jabberwocky [SEM × CV × LAT: *F*(1, 18) = 8.51, *p* = 0.009]. Further, a non-significant trend suggested that the posterior positivity was reduced in amplitude for Jabberwocky in musicians [SEM × CV: *F*(1, 18) = 3.45, *p* = 0.080].

#### Expertise effects in language

The early AN elicited by correctly-detected phrase-structure violations in English sentences was left-lateralized in musicians but not in non-musicians [two anterior-most rows GP × CV × AP × HEM: *F*(1, 38) = 5.66, *p* = 0.022; most anterior row, GP × CV × HEM: *F*(1, 38) = 3.96, *p* = 0.054]. Musical expertise did not modulate the amplitude or distribution of the late AN or posterior positivity with English (*p*s > 0.1).

There was no indication that musical expertise modulated the amplitude or distribution of the AN measured in either time window with Jabberwocky (*p*s > 0.3). Musical expertise did not modulate the amplitude of the posterior positivity with Jabberwocky (*p* > 0.4), but this effect was more medially distributed in non-musicians [GP × CV × LAT: *F*(1, 37) = 5.69, *p* = 0.022]. Musical expertise did not modulate the amplitude reduction of the posterior positivity for Jabberwocky (*p* > 0.9). With all participants as a single group, the posterior positivity was marginally smaller with Jabberwocky compared to English [SEM × CV: *F*(1, 37) = 3.87, *p* = 0.057].

### Language discussion

Consistent with previous research, phrase-structure violations in English and semantically impoverished Jabberwocky sentences elicited a biphasic ERP response in both participant groups. Musical expertise and semantic richness affected the AN and P600 in distinct ways.

#### Effects of semantic richness

The similar latency and amplitude of the AN observed in the English and Jabberwocky conditions is in line with the argument that the AN selectively indexes syntactic processing. However, sentence type did affect the distribution of the AN indicating that semantic information influences syntactic processing. Yamada and Neville (2007) reported differences in the extent to which the AN was observed over more temporal regions for English and Jabberwocky sentences; in the current study the AN had a more laterally extended distribution for English sentences. Differences in the extent to which the AN extended to the most lateral sites cannot be attributed to differences in P600 amplitude that might overlap with the AN. However, differences in the way the distribution of the AN was affected by sentence type in these two studies suggests that the broader semantic context of the experiment or the modality in which stimuli were presented impacts the way in which semantic content modulates early syntactic processing.

The reduction in P600 amplitude for phrase-structure violations in Jabberwocky sentences was small but present across participant groups. Using simple sentences that allow for accurate detection of syntactic violations in Jabberwocky sentences may allow for some attempts at syntactic repair even with sentences that have reduced semantic content. Presenting both the Jabberwocky and English version of the same sentences within a session may further reduce the processing differences between the sentence types. However, a reduced or absent P600 indicates that semantic content influences syntactic revision and repair processes. Specifically, with Jabberwocky sentences, for which rescuing the meaning of ungrammatical sentences is less likely, less effort is dedicated to these processes (Münte et al., 1997; Kaan et al., 2000).

#### Effects of musical expertise

Musical expertise was found to modulate the lateralization of the AN for English sentences. The effects of musical expertise on early linguistic syntactic processing were remarkably similar to the effects of linguistic proficiency reported by Pakulak and Neville (2010); greater musical proficiency resulted in a more focally distributed and left-lateralized AN. This training transfer effect between music and language complements those reported in a previous study with children. Jentschke and Koelsch (2009) found that musically trained children show a more adult-like AN to linguistic violations than their untrained counterparts. The results of Jentschke and Koelsch (2009) could be interpreted as either a speeding up of the typical developmental timeline or a qualitative change in early linguistic syntactic processing as a function of musical expertise. Our findings suggest that the training transfer effect observed in children represents a qualitative change; if musical experience were only speeding up linguistic development, no training transfer effect would be observed in adults.

Without linguistic proficiency measures, the possibility that the current effects reflect linguistic rather than musical proficiency cannot be ruled out. However, there were no group differences in performance on the linguistic grammaticality judgment task. Further, in contrast to early syntactic processing, the effects of musical expertise do not resemble the effects of linguistic proficiency on later syntactic processing. Pakulak and Neville (2010) reported an increase in P600 amplitude as a function of linguistic proficiency; we found no differences in P600 amplitude as a function of musical expertise. Since the same sentences and a similar task were used in both studies, this pattern of results suggests a difference between the effects of linguistic and musical proficiency on syntactic revision and repair processes.

### Music results

#### Music behavioral results

As shown in Figure 3, both groups of participants detected harmonic violations in musical phrases and showed better performance with blatant than subtle out-of-key substitutions. Non-musicians’ performance was above chance [blatant: *d*′ = 2.22, *SD* = 0.84, *t*(19) = 11.86, *p* < 0.001; subtle: *d*′ = 0.63, *SD* = 0.34, *t*(19) = 8.32, *p* < 0.001] and better for blatant violations, *t*(19) = 9.54, *p* < 0.001. Musicians’ performance was above chance for both types of violations [blatant: *d*′ = 3.29, *SD* = 1.27, *t*(19) = 11.57, *p* < 0.001; subtle: *d*′ = 1.87, *SD* = 0.99, *t*(19) = 8.45, *p* < 0.001] and better for blatant ones, *t*(19) = 5.86, *p* < 0.001. Musicians were better than non-musicians at detecting both blatant, *t*(38) = 3.14, *p* = 0.004, and subtle violations, *t*(38) = 5.28, *p* < 0.001.

#### Music ERP results

**Figure 5:**
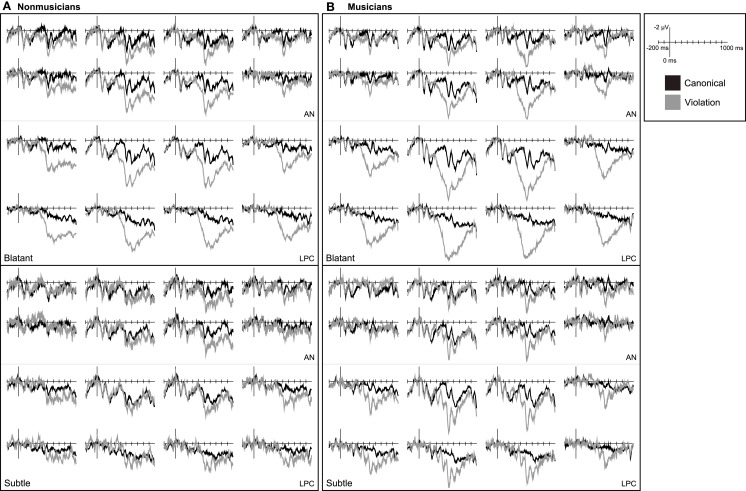
**Music ERPs**. Grand average ERP waveforms elicited by critical chords in canonical and violation contexts measured in **(A)** non-musicians and **(B)** musicians. Data for chord substitutions from distantly related keys are shown on the top and from closely related keys on the bottom.

In non-musicians correctly-detected harmonic violations elicited a late posterior positivity but no AN (Figure 5). The AN was absent 150–300 ms in response to chord substitutions from both distantly and closely related keys (*p*s > 0.2). Blatant harmonic violations elicited a large posterior positivity 400–950 ms, *F*(1, 19) = 24.92, *p* < 0.001. This effect was larger over posterior and medial regions [CV × LAT: *F*(1, 19) = 17.79, *p* < 0.001; CV × AP: *F*(2, 38) = 3.67, *p* = 0.055; CV × AP × LAT: *F*(2, 38) = 4.70, *p* = 0.020]. Subtle harmonic violations also elicited a posterior positivity 400–950 ms, but this effect was limited to the right-hemisphere [all regions CV × HEM: *F*(1, 19) = 3.71, *p* = 0.069; right CV: *F*(1, 19) = 4.50, *p* = 0.047]. The positivity elicited by subtle violations was smaller and more right-lateralized [DIS × CV: *F*(1, 19) = 5.87, *p* = 0.026; DIS × CV × HEM: *F*(1, 19) = 4.91, *p* = 0.039; DIS × CV × LAT: *F*(1, 19) = 11.04, *p* = 0.004].

In musicians listening to musical phrases, blatant violations elicited a right-lateralized AN and both blatant and subtle violations elicited a late posterior positivity (Figure 5). For blatant violations the AN 150–300 ms after onset was observed only at right, central sites [all regions CV × HEM: *F*(1, 19) = 4.86, *p* = 0.040; right CV: *F*(1, 19) = 4.22, *p* = 0.054; two least-anterior rows right CV: *F*(1, 19) = 6.65, *p* = 0.018]. Blatant violations also elicited a posterior positivity 400–950 ms after onset, *F*(1, 19) = 64.91, *p* < 0.001. This effect was larger at more posterior and medial sites [CV × AP: *F*(2, 38) = 20.80, *p* < 0.001; CV × LAT: *F*(1, 19) = 26.73, *p* < 0.001; CV × AP × LAT: *F*(2, 38) = 7.05, *p* = 0.004; CV × LAT × HEM: *F*(1, 19) = 4.27, *p* = 0.053]. Subtle harmonic violations did not elicit an AN (*p*s > 0.2), but did elicit a posterior positivity 400–950 ms, *F*(1, 19) = 6.06, *p* = 0.024. The posterior positivity in response to subtle violations was larger over posterior and medial regions [CV × AP: *F*(2, 38) = 3.11, *p* = 0.085; CV × LAT: *F*(1, 19) = 5.84, *p* = 0.026; CV × AP × HEM: *F*(2, 38) = 7.83, *p* = 0.005; CV × AP × LAT × HEM: *F*(2, 38) = 2.95, *p* = 0.068]. A non-significant trend suggested that the posterior positivity was larger in response to blatant violations [DIS × CV: *F*(1, 19) = 3.62, *p* = 0.072].

#### Expertise effects in music

Although blatant harmonic violations elicited a right, central AN 150–300 ms in musicians but not in non-musicians, musical expertise did not modulate the effect (*p* > 0.15). Further, the RAN was evident with participants collapsed into a single group, *F*(1, 38) = 7.13, *p* = 0.011. Likewise, expertise did not modulate the posterior positivity to blatant violations (*p*s > 0.2). The positivity elicited by subtle violations was more medially distributed in musicians [GP × CV × LAT: *F*(1, 38) = 5.64, *p* = 0.023] and was more right-lateralized in non-musicians [GP × CV × HEM: *F*(1, 38) = 4.08, *p* = 0.051]. However, expertise did not modulate the amplitude of the posterior positivity where it was observed in non-musicians (right only, *p* > 0.8).

### Music discussion

#### Anterior negativity

The distribution and latency of the AN to harmonic violations observed in musicians is somewhat similar to what has previously been reported for syntactic processing of music. The right-temporal distribution is shared with other studies that included harmonic violations at unpredictable times (Patel et al., 1998; Koelsch and Mulder, 2002). However, Steinbeis et al. (2006) reported a frontally distributed AN for harmonic violations at unpredictable times, so the right-temporal distribution cannot be cleanly attributed to the inability of listeners to prepare for violations before they occur. In the current study, the AN had the early latency typically described for an ERAN (e.g., Koelsch et al., 2000). The onset was earlier than that observed in studies when the timing of violations was unpredictable. It has been suggested that the early onset of the ERAN is dependent on low rhythmic complexity (Koelsch, 2009); the musical phrases in the current study were isochronous, providing tentative support for this argument.

In contrast to prior studies, an AN was not evident in non-musicians when this group was considered alone. Further, the AN observed in musicians was smaller and more topographically restricted than what is typically reported. The lack of interaction with music-expertise group for the AN suggests a lack of power to detect a small effect in non-musicians rather than a fundamental difference in how the syntactic violations were processed in the two groups. The small and variable AN can be attributed to the variability in relative function of the target chords. Variability in the relative function of grammatical, in-key and harmonically appropriate chords creates variability in harmonic expectedness (Krumhansl et al., 1982; Bharucha and Krumhansl, 1983). Less harmonically expected in-key chords have been shown to elicit an AN (Steinbeis et al., 2006; Koelsch et al., 2007; Carrión and Bly, 2008). In the current study, the grammatical but less harmonically expected in-key chords in the canonical conditions may have elicited an AN, decreasing the difference between the grammatical and ungrammatical conditions.

#### Late positive component

Harmonic violations elicited a posterior LPC 400–950 ms in both musicians and non-musicians, replicating the results of prior music syntactic studies employing task-relevant violations in unfamiliar melodies and chords (e.g., Besson and Faïta, 1995; Patel et al., 1998). Musical expertise did not modulate LPC amplitude for either the blatant or subtle violation conditions. This pattern differs from what has been found for ungrammatical single notes, but is identical to Regnault et al. (2001)’s findings for ungrammatical chords. Musical expertise may modulate LPC amplitude for harmonic violations in monophonic but not polyphonic phrases because expertise is required to extract the implied harmonic information from monophonic phrases but is available to both musicians and non-musicians listening to polyphonic phrases.

The LPC elicited by blatant violations was larger in amplitude than that elicited by subtle violations in both musicians and non-musicians, similar to what has been reported for expectancy violations in melodies (Besson and Faïta, 1995; Brattico et al., 2006) and chord progressions (Janata, 1995; Patel et al., 1998; Carrión and Bly, 2008). The type of expectancy violations differs across these studies, but in all cases the LPC was larger for the more difficult to integrate event. In the current study, chords from more distantly related keys were more difficult to integrate than chords from more closely related keys. Difficulty of integrating the violation also affected the distribution of the LPC. The differences in distribution may reflect differing contributions of pitch commonality and chord function expectancy for the blatant and subtle violations. It has been suggested that processing of these two types of harmonic incongruity makes distinct contributions to the LPC (Regnault et al., 2001). The blatant violations had much less probable chord functions than the grammatical chords they replaced and contained between one and three out-of-key notes; the subtle violations had only somewhat less probable chord functions and always contained one out-of-key note. However, as was true for the differences in AN distribution in response to syntactic violations in English and Jabberwocky sentences, in the current study the LPC distribution differences were observed along the medial-lateral axis rather than the anterior-posterior axis as previously reported (Regnault et al., 2001).

### Cross-domain comparisons

#### Behavioral data

Overall, listeners were better at detecting syntactic violations in sentences than in musical phrases (Figure 3). Non-musicians’ performance was better with the more difficult of the language conditions, Jabberwocky, than with the easier of the music conditions, blatant harmonic violations, *t*(19) = 2.39, *p* = 0.027. For musicians, performance was indistinguishable in these two conditions (*p* < 0.6) reflecting this groups better ability to detect harmonic violations.

#### ERP comparisons

In non-musicians an AN was observed in response to phrase-structure violations (100–250 ms over the two anterior-most rows of electrodes and 300–500 ms over the anterior-most electrodes) but not harmonic violations (150–300 ms). The P600 elicited by phrase-structure violations (200–750 ms) fell in an overlapping time window with the LPC elicited by harmonic violations (400–950 ms). The P600 was evident 200–400 ms, but the LPC was not [DOM × CV: *F*(1, 19) = 22.45, *p* < 0.001]. In a direct comparison of the two posterior positivities measured in their different time windows, the P600 had a more posterior maximum and was more medially distributed than the LPC [DOM × CV × AP: *F*(2, 38) = 21.87, *p* < 0.001; DOM × CV × LAT: *F*(1, 19) = 14.09, *p* = 0.001].

In musicians an AN was observed in response to phrase-structure violations (100–250 and 300–500 ms) and blatant harmonic violations (150–300 ms). Effects of grammaticality on mean amplitude 100–150 ms for language but not music indicate the language AN had an earlier onset [English, two anterior-most rows CV: *F*(1, 19) = 9.90, *p* = 0.005; Jabberwocky, two anterior-most rows CV: *F*(1, 18) = 7.02, *p* = 0.016; blatant, two least-anterior rows over the right-hemisphere: *p*s > 0.3]. The AN elicited by blatant harmonic violations was more right-lateralized and extended over more central regions than the early AN observed with language stimuli [English and blatant DOM × CV × HEM: *F*(1, 19) = 13.89, *p* = 0.001 and DOM × CV × AP: *F*(2, 38) = 16.49, *p* < 0.001 and DOM × CV × LAT: *F*(1, 19) = 6.98, *p* = 0.016; Jabberwocky and blatant DOM × CV × HEM: *F*(1, 18) = 6.40, *p* = 0.021 and DOM × CV × AP: *F*(2, 36) = 12.61, *p* = 0.001]. As was true for non-musicians, the P600 elicited by phrase-structure violations (200–750 ms) fell in an overlapping time window with the LPC elicited by harmonic violations (400–950 ms). The P600 was evident 200–400 ms, *F*(1, 18) = 14.79, *p* = 0.001. The LPC was evident 200–400 ms, but only for blatant harmonic violations over medial electrodes, *F*(1, 19) = 4.91, *p* = 0.039. A non-significant trend suggested that the P600 was larger than the LPC 200–400 ms over all electrodes [DOM × CV: *F*(1, 18) = 3.91, *p* = 0.063]. In a comparison of the two posterior positivities measured in their different time windows, the P600 observed with speech was more right-lateralized than the LPC elicited in music [DOM × CV × HEM: *F*(1, 18) = 7.96, *p* = 0.011]. Non-significant trends suggested that the LPC was larger than the P600, especially over medial electrodes [DOM × CV: *F*(1, 18) = 3.67, *p* = 0.071; DOM × CV × LAT: *F*(1, 18) = 3.03, *p* = 0.099].

#### Cross-domain discussion

The AN elicited by blatant harmonic violations in musicians onset slightly later than the AN elicited by phrase-structure violations, replicating previous reports (e.g., Friederici et al., 1993; Koelsch et al., 2000). Additionally, the AN elicited by harmonic violations was right-lateralized and more centrally distributed, replicating previous within-subjects comparisons (Patel et al., 1998). These findings provide further evidence that the mechanisms underlying early syntactic processing in language and music are not identical.

The late positivities elicited by phrase-structure and harmonic violations were not identical in the current study, in contrast to previous reports (Patel et al., 1998), suggesting that late syntactic processing of language and music rely on at least partially distinct mechanisms. However, there have also been previous reports of within-domain latency and distribution differences for late positivities (Regnault et al., 2001; Gouvea et al., 2010). Further, in the current study the P600 differed for English and Jabberwocky in musicians and the LPC differed for blatant and subtle music violations. Domain may not be a critical feature that defines the timing and distribution of P600s and LPCs, and is certainly not the only one.

### General discussion

The current results are consistent with those of previous within-domain studies of syntactic processing in language and music; task-relevant phrase-structure and harmonic violations elicit anterior negativities and later posterior positivities. Contrary to predictions, the AN elicited by harmonic violations was only evident in musicians. It is likely that, as reported in previous studies, the music AN was present in non-musicians but was smaller in amplitude compared to that in experts. Musical expertise did not affect LPC amplitude, though it did modulate the distribution of this effect. The current results are largely consistent with previous research directly comparing ERP indices of linguistic and music syntactic processing within the same adults (Patel et al., 1998); harmonic violations elicited a more right-lateralized AN than phrase-structure violations. However, unlike reported by Patel et al. (1998), the late positivities elicited by phrase-structure and harmonic violations differed in both latency and distribution.

The primary goal of the current study was to test the hypothesis that musical expertise modulates cortical organization of linguistic syntactic processing; the ERP results support this hypothesis. Musical expertise resulted in a more focally distributed and left-lateralized AN in response to syntactic violations in language. This result adds to a growing body of evidence that the mechanisms supporting linguistic and music syntactic processing interact as posited by the SSIRH (Patel, 2003; Koelsch et al., 2005; Steinbeis and Koelsch, 2008; Fedorenko et al., 2009; Jentschke and Koelsch, 2009; Slevc et al., 2009; Hoch et al., 2011; Maidhof and Koelsch, 2011). The cross-domain effect of music-expertise on language processing provides electrophysiological evidence that syntactic processing in these domains is supported, in part, by domain-general neural mechanisms. For musical training to modulate the cortical organization of linguistic syntactic processing, linguistic syntactic processing in musically untrained individuals must rely on cortical regions that change during the acquisition of musical expertise. Given prior evidence that linguistic and musical proficiency modulate neural substrates of within-domain early syntactic processing (Koelsch et al., 2002a, 2007; Pakulak and Neville, 2010), it seems likely that as right-hemisphere neural structures become more specialized for music processing in musicians, left-hemisphere structures become more specialized for language processing in these same individuals.

The precise nature of the relationship between shared neural substrates and the functions they support remains an open question. However, the current results suggest that a domain-general neural resource serves as a node recruited as part of multiple domain-specific functions. In the general population, syntactic processing of both language and music draw on the computations carried out by both left- and right-hemisphere structures. Musical training results in better processing of music syntax as indexed by behavioral measures and specialization of right-hemisphere regions for music syntactic processing as indexed by the early right-lateralized ERP effects in response to syntactic violations. The right-hemisphere neural resource is no longer as effective for supporting the non-identical syntactic processing that is carried out in the language domain. As such, the left-hemisphere regions that have been unaffected by music training come to dominate linguistic syntactic processing. Critically, the left-hemisphere regions may also become more specialized, resulting in linguistic syntactic proficiency that is at least equivalent to that observed in musically untrained individuals, but supported by fewer, more efficient, neural regions. Although the behavioral measure of linguistic syntactic processing employed in the current study may not have been sensitive enough to detect subtle differences in linguistic proficiency, the lack of any difference in performance with the language stimuli in musically trained and untrained listeners suggests expertise can result in better within-domain performance without performance costs in other domains that also show cortical reorganization.

## Conflict of Interest Statement

The authors declare that the research was conducted in the absence of any commercial or financial relationships that could be construed as a potential conflict of interest.

## Appendix

### Musical items

#### Blatant condition

Phrases were derived from the 10 relative progressions shown below. Each progression was spelled out in the three indicated key pairs. The canonical phrases had their fourth (key pair 1), fifth (key pair 2), or sixth (key pair 3) chords switched to create two violation phrases.

**Table:**

Parent progression							Key pair 1	Key pair 2	Key pair 3
I	IV	V	iii	ii	V	I	C and C♯	F and F♯	A and B ♭
I	V	I^6^	I	ii	iii	I	F♯ and G	C♯ and D	B ♭ and B
I	iii	ii	vii°	I	IV	I	D and E ♭	B and C	G and A ♭
I	V	vi	ii	iii	ii	I	C and C♯	E ♭ and E	A ♭ and A
I	vi	iii	IV	V	IV	I^(8va)^	C♯ and D	A and B ♭	E and F
I	iii	ii	V	vi	vii°	I^(8va)^	B ♭ and B	D and E ♭	F and F♯
I	V	I^(8vb)^	V	I	V	I^(8vb)^	F♯ and G	B and C	E ♭ and E
I	ii	iii	IV	iii	ii	I	E ♭ and E	G and A ♭	B and C
I	vii°	vi	V	vi	vii°	I	C♯ and D	F and F♯	A ♭ and A
I	IV	vi	ii	V	vii°	I	E ♭ and E	A and B ♭	G and A ♭

#### Subtle condition

Phrases were derived from 30 progression pairs. In 15 of the pairs a subdominant (IV) chord occurred in one progression and a supertonic (ii) chord occurred in the corresponding position (fourth, fifth, or sixth) of the other. The other 15 pairs had a dominant (V) and a mediant (iii) chord in these critical positions.

**Table:**

IV/ii Progression pairs							Key	V/iii Progression pairs							Key
Manipulation at fourth position								Manipulation at fourth position							
I	ii	iii	IV	vi	V	I	A	I	ii	iii	V	vi	V	I	A
I	V	iii	ii	iii	IV	I	C	I	IV	ii	iii	IV	iii	I	C
I	V	vi	IV	V	ii	I	F	I	iii	ii	V	IV	iii	I	C
I	ii	iii	ii	IV	iii	I	A ♭	I	iii	IV	iii	V	IV	I	E ♭
I	V	iii	IV	V	IV	I	B ♭	I	iii	IV	V	vi	V	I	B
I	V	vi	ii	V	IV	I	C♯	I	vii°	vi	iii	ii	iii	I	D
I	iii	ii	IV	V	vi	I	E	I	IV	iii	V	IV	V	I	E
I	iii	V	ii	iii	IV	I	G	I	V	IV	iii	vi	V	I	G
I	vi	ii	IV	iii	ii	I	B	I	IV	ii	V	vi	IV	I	F
I	IV	iii	ii	iii	ii	I	D	I	ii	IV	iii	V	IV	I	A ♭
Manipulation at fifth position								Manipulation at fifth position							
I	V	vi	iii	IV	ii	I	E ♭	I	IV	ii	iii	V	vi	I	B ♭
I	IV	V	IV	ii	V	I	F♯	I	V	vi	V	iii	IV	I	C♯
I	ii	iii	V	IV	V	I	E	I	iii	ii	IV	V	iii	I	B
I	IV	iii	IV	ii	iii	I	G	I	IV	iii	ii	iii	ii	I	D
I	iii	V	vi	IV	iii	I	F	I	IV	iii	vi	V	iii	I	G
I	IV	vi	V	ii	IV	I	A ♭	I	iii	V	ii	iii	IV	I	B ♭
I	vi	iii	ii	IV	V	I	F♯	I	IV	iii	ii	V	ii	I	E ♭
I	vii°	vi	V	ii	IV	I	A	I	vii°	vi	V	iii	ii	I	F♯
I	iii	ii	V	IV	iii	I	A ♭	I	ii	iii	IV	V	IV	I	A ♭
I	${\text{I}}_{\text{4}}^{\text{6}}$	V	vi	ii	${\text{IV}}_{\text{4}}^{\text{6}}$	I	B	I	ii	IV	vi	iii	V	I	B
Manipulation at sixth position								Manipulation at sixth position							
I	vi	V	IV	iii	IV	I	E ♭	I	iii	ii	IV	iii	V	I	C
I	IV	V	IV	vi	ii	I	F♯	I	vi	V	IV	ii	iii	I	E ♭
I	IV	iii	IV	ii	IV	I	F♯	I	ii	iii	IV	vi	V	I	C♯
I	vi	V	IV	iii	ii	I	A	I	iii	vi	V	vi	iii	I	E
I	V	IV	I	V	IV	I	D	I	IV	iii	vi	ii	V	I	D
I	IV	iii	I	V	ii	I	F	I	V	IV	iii	ii	iii	I	F
I	V	iii	I	ii	IV	I	G	I	vii°	vi	IV	iii	V	I	A
I	V	IV	iii	IV	ii	I	B ♭	I	ii	iii	IV	ii	iii	I	C
I	IV	iii	ii	I	IV	I	C♯	I	iii	V	ii	IV	V	I	B ♭
I	iii	V	IV	iii	ii	I	E	I	IV	iii	vi	V	iii	I	C♯
